# Early Postoperative Analgesic Evaluation of Intravenous Lidocaine Infusion or a Combination of Intraperitoneal and Incisional Lidocaine Splash in Female Dogs Undergoing Ovariohysterectomy

**DOI:** 10.3390/vetsci12121116

**Published:** 2025-11-24

**Authors:** Wanwisa Chaoum, Piyasak Wipoosak, Suvaluk Seesupa, Benedict Duncan X. Lascelles, Supranee Jitpean, Naruepon Kampa, Thanikul Srithunyarat

**Affiliations:** 1 Division of Surgery, Faculty of Veterinary Medicine, Khon Kaen University, Khon Kaen 40002, Thailand; wanwisa.cha@kkumail.com (W.C.); suvalukse@kku.ac.th (S.S.); supraneeji@kku.ac.th (S.J.); naruepon@kku.ac.th (N.K.); 2 Veterinary Teaching Hospital, Faculty of Veterinary Medicine, Khon Kaen University, Khon Kaen 40002, Thailand; piyawi@kku.ac.th; 3 Translational Research in Pain Program, Comparative Pain Research and Education Centre, Department of Clinical Sciences, College of Veterinary Medicine, North Carolina State University, Raleigh, NC 27606, USA; dxlascel@ncsu.edu; 4 Center for Translational Pain Research, Department of Anesthesiology, Duke University, Durham, NC 27710, USA; 5 Thurston Arthritis Center, University of North Carolina, Chapel Hill, NC 27599, USA

**Keywords:** canine, local anesthetics, locoregional block, intravenous infusion, multimodal pain management, pain assessment, spaying

## Abstract

Combined intraperitoneal and incisional block provides effective perioperative analgesia but is limited in clinical practice. Intravenous lidocaine offers an alternative, although its analgesic efficacy remains controversial. This study compared the postoperative analgesic efficacy of lidocaine intravenous infusion with combined intraperitoneal and incisional lidocaine splash in dogs undergoing ovariohysterectomy. Thirty female dogs were randomized to intravenous infusion (IV), intraperitoneal and incisional splash (IP + SP), or control (C). Anesthesia included intramuscular acepromazine (0.03 mg/kg), intravenous propofol (4–6 mg/kg), isoflurane maintenance, and fentanyl (2 µg/kg) intravenous pre-incision. Group IV received intravenous lidocaine 2 mg/kg and then 50 μg/kg/min infusion. Group IP + SP received lidocaine 4 mg/kg intraperitoneally intraoperatively and 2 mg/kg as an incisional splash before skin closure. Controls received saline. Postoperative pain was assessed using the Glasgow Composite Measure Pain Scale—Short Form and analyzed using a linear mixed model. At 60 min, pain scores were 1.2 ± 0.4 (IP + SP), 1.6 ± 0.7 (IV), and 3.0 ± 2.8 (C) and at 120 min 1.3 ± 0.5, 1.4 ± 0.7, and 2.3 ± 0.8, respectively. Both treatments had significantly lower scores than controls (*p* = 0.004); IV and IP + SP did not differ. Therefore, intravenous lidocaine infusion may be an option for early postoperative analgesia in canine ovariohysterectomy.

## Simple Summary

Ovariohysterectomy is a common surgical procedure in dogs, associated with a moderate level of pain. A combination of intraperitoneal and incisional block techniques is recommended as locoregional anesthesia in a multimodal analgesia strategy to provide effective perioperative pain control. However, the implementation of these techniques in clinical practice may be limited. Intravenous lidocaine infusion has been used for perioperative pain management in dogs. This technique may offer a technically straightforward alternative that can be readily implemented in general practice settings. This study aimed to investigate the analgesic efficacy of intravenous lidocaine infusion in comparison with a combination of lidocaine intraperitoneal and incisional block techniques in dogs undergoing ovariohysterectomy. The results demonstrated that both techniques provided effective postoperative analgesia, as indicated by low pain scores and no requirement for rescue analgesia. No significant differences in analgesic efficacy were observed between intravenous infusion and locoregional block techniques using lidocaine. Therefore, intravenous lidocaine infusion may be an alternative protocol for pain management for up to 2 h in female dogs undergoing ovariohysterectomy.

## 1. Introduction

Ovariohysterectomy (OVH) is a commonly performed surgical procedure in dogs associated with moderate pain intensity [1]. Surgical intervention can trigger nociceptive and inflammatory pathways that induce neuroplasticity within the spinal cord, leading to central sensitization through activation of excitatory neurotransmitters and multiple receptor systems in the dorsal horn [2]. Effective prevention of both peripheral and central sensitization is crucial for acute postoperative pain control. Therefore, optimal perioperative pain management is essential for enhancing recovery and minimizing postoperative complications. Inadequate pain management may result in delayed recovery and a prolonged return to normal function [3].

For canine OVH, current pain management guidelines recommend the use of locoregional anesthesia, specifically a combination of intraperitoneal and incisional block techniques, as part of a multimodal analgesic approach [4,5]. Local anesthetics are commonly used due to their analgesic efficacy, which can reduce the requirements for inhalant anesthetics, shorten recovery periods, and improve patient comfort [6,7,8]. Despite these advantages, several practical challenges and concerns regarding potential adverse effects persist, such as tissue irritation, nerve injury, or systemic toxicity [9,10,11]. As a result, the use of these techniques remains limited in many veterinary settings.

Lidocaine, a sodium channel blocker, is unique among local anesthetics used for pain management because it can be administered systemically in dogs [12]. The mechanism of action of intravenous lidocaine appears to be complex and involves multiple pathways [13,14]. Evidence suggests that systemic lidocaine may exert effects not only through sodium channel blockade but also via potential interactions with N-methyl-D-aspartate (NMDA) receptors in the central nervous system, particularly within the spinal cord [15]. These receptors are involved in pain modulation and the development of central sensitization related to chronic pain states [16]. Moreover, systemic lidocaine has been reported to have anti-inflammatory effects [17,18]. While systemic lidocaine is considered useful, common adverse effects of intravenous lidocaine, such as vomiting, tremors, confusion, and seizures, have been reported [11,19]. Furthermore, the antinociceptive effect and acute postoperative pain control provided by systemic lidocaine in dogs remain inconsistent, with some studies reporting conflicting outcomes [20,21,22].

Lidocaine infusion is used for analgesic management in human medicine, particularly in conditions such as chronic neuropathic pain [23]. In small animal medicine, intravenous lidocaine infusion is used for managing cardiac arrhythmia in conscious dogs [24] and as an adjunct during inhalation anesthesia due to its anesthetic-sparing effects. Several studies have shown that intravenous lidocaine infusion reduces the minimum alveolar concentration of sevoflurane [20,25,26,27] and isoflurane [28,29,30,31] in a dose-dependent manner. To the authors’ knowledge, the analgesic effects of intravenous lidocaine infusion, compared to locoregional lidocaine via a combination of intraperitoneal and incisional splash blocks, have not been previously reported. This study aims to evaluate the early postoperative analgesic efficacy of intravenous lidocaine infusion in comparison with a combination of lidocaine intraperitoneal and incisional splash block techniques in dogs undergoing OVH. We hypothesized that intravenous lidocaine infusion would reduce early postoperative pain scores comparable to the combination of lidocaine intraperitoneal and incisional splash block techniques in dogs undergoing OVH.

## 2. Materials and Methods

This prospective, randomized, blinded, clinical study was approved by the Institutional Animal Care and Use Committee of Khon Kaen University, Thailand (IACUC-KKU-53/66 and 28/66). All owners provided written informed consent, and the study adhered to the ARRIVE guidelines [32].

### 2.1. Dogs

Thirty client-owned healthy female dogs were scheduled for routine OVH at the Veterinary Teaching Hospital, Faculty of Veterinary Medicine, Khon Kaen University. Dogs weighing between 4 and 25 kg and aged 6 months to 6 years were enrolled in this study. All dogs were included if they were considered healthy based on physical examination, abdominal ultrasonography, complete blood count, and serum chemistry profile (total protein, albumin, creatinine, and alanine transaminase), and were categorized as American Society of Anesthesiologists physical status class I or II (ASA I or II). Dogs were excluded if they exhibited aggressive behavior or systemic illness. Food was withheld for 4–6 h preoperatively, and water was allowed until arrival at the hospital.

### 2.2. Study Design

Thirty dogs were allocated by block randomization, with blocks of 6, to three treatment groups (10 dogs per group): intravenous lidocaine infusion (IV) group, intraperitoneal and incisional lidocaine splash (IP + SP) group, and control (C) group. The IV group, lidocaine was administered intravenously over 2 min at a dose of 2 mg/kg (2% Lidocaine hydrochloride injection; GPO Pharmaceuticals, Bangkok, Thailand), followed by a constant rate infusion (CRI) of lidocaine at 50 μg/kg/min. The lidocaine (300 mg) was diluted in 500 mL lactated Ringer’s solution (lactated Ringer’s Solution; GHP, Pathum Thani, Thailand) and CRI using the fluid rate of 5 mL/kg/h. The IP + SP group received 4 mg/kg of lidocaine diluted with 0.9% NaCl (NSS, General Hospital Products Public CO., Pathum Thani, Thailand) to achieve 0.6 mL/kg for administration as an intraperitoneal (IP) splash during surgery (final dilution of 0.67% lidocaine), and an additional 2 mg/kg of lidocaine was diluted with an equal volume of 0.9% NaCl (final dilution of 1% lidocaine) for administration as an incisional splash (SP) prior to skin closure. Lidocaine was used according to the designated treatment group, and 0.9% NaCl in the same volume was used in the other. Specifically, the IV group received lidocaine intravenously and infusion; 0.9% NaCl was used in place of IP and SP. The IP + SP group received intravenous 0.9% NaCl and lactated Ringer’s solution for infusion, and lidocaine for IP and SP. The C group received 0.9% NaCl in equivalent volumes and via the same routes of administration as the other groups, and lactated Ringer’s solution for infusion. The infusions were discontinued at the end of the surgery. This study was conducted by the same surgeon (T.S.), anesthetist (W.C.), and pain assessor (W.C.), all of whom were blinded to treatment assignments. The trial coordinator (P.W.), who did not participate in assessing the dogs, was responsible for group allocation and drug preparation on all occasions.

### 2.3. Anesthesia and Ovariohysterectomy

Prior to premedication, baseline physiologic variables (T0) were recorded for all dogs, including heart rate (HR) assessed by auscultation with stethoscope, respiratory rate (*f*_R_) determined by observation of chest movement, and body temperature measured with a rectal thermometer, and noninvasive arterial blood pressure (NIBP), including systolic (SAP), mean (MAP), and diastolic (DAP) arterial pressure, was measured using an oscillometric device (BP-AccugaurdTM; Vmed Technology, Mill Creek, WA, USA) with an appropriately sized cuff (width approximately 30–40% of limb circumference) placed above the carpus. Additionally, preoperative sedation [33] and pain scores using the Glasgow Composite Measure Pain Scale—Short Form (CMPS-SF) [34] were assessed.

All dogs were premedicated with acepromazine (0.03 mg/kg) intramuscularly (Combistress, Kela N.V., Hooogstraten, Belgium). Ten to 15 min after premedication, a venous catheter of size 22 or 24 gauge was placed aseptically into a cephalic vein to initiate fluid therapy with lactated Ringer’s solution and preoxygenated for 5–7 min before induction. Dogs were administered prophylaxis antibiotic treatment with 25 mg/kg cefazolin intravenously (Cefaben^®^, L.B.S. Laboratory Ltd., Bangkok, Thailand). General anesthesia was induced with intravenous propofol (1% Profol^TM^, Baxter Pharmaceuticals India Private Limited, Gujarat, India) titrated to effect with a dose range of 4–6 mg/kg until an orotracheal tube was possibly inserted and secured in place. Dogs were placed in dorsal recumbency, and the surgical site was prepared using aseptic techniques. Anesthesia was maintained using a rebreathing circuit with isoflurane (Terrell, Piramal Critical Care Inc., Bethlehem, PA, USA) delivered in pure oxygen, allowing spontaneous breathing throughout the surgery. Determination of the isoflurane vaporizer setting to maintain surgical depth and match noxious stimulation of anesthesia was adjusted by the same anesthetist (W.C.) based on clinical signs, including ventromedial position of eyeballs, absence of palpebral reflex, and slight jaw tone (T1). A forced-air warming system (Bair Hugger 505; Augustine Medical, Eden Prairie, MN, USA) was used to maintain body temperature at 37–38 °C. Before skin incision, dogs received 2 µg/kg of fentanyl (Fentanyl-Hameln 50 µg/mL, Siam Bioscience Co., Nonthaburi, Thailand) intravenously over a minute. Subsequently, the intravenous solution (lidocaine in the IV group or 0.9% NaCl in the IP + SP and C groups) was administered over two minutes by hand through the same venous access using a 3-way stopcock prior to skin incision. A CRI was then maintained via infusion pump throughout the surgery and discontinued at the end of the surgical procedure upon skin closure.

The OVH procedure was approached via a caudal midline celiotomy [35] performed by the same surgeon (T.S.). Each dog received all routes of the prepared solutions (IV, IP, and SP) according to their assigned groups (lidocaine as a treatment and 0.9% NaCl as a control instead of lidocaine in equivalent volume). Following the approach into the abdominal cavity (T2), the IP solution was administered and separated into two portions. Before ligation, the first portion (0.2 mL/kg), one-third of the solution, was splashed onto each ovarian pedicle (right and then left), cervix, and the uterine body, respectively, allowing for a minimum contact time of one minute. After removal of both ovaries and uterus (T3–T5), the second portion of the IP solution (0.4 mL/kg) was then splashed into the abdominal cavity before abdominal closure. Once the linea alba was closed, the SP solution was applied by dripping onto the subcutaneous tissue at the incision site. The skin was then lifted and held to allow a contact time of 2 min. The subcutaneous tissue and skin were then closed using a simple interrupted suture for the linea alba, subcutaneous tissue, and skin layer (T6). Anesthesia was discontinued after the last skin suture. Upon observing the swallowing reflex, dogs were extubated and transported to the quiet recovery room.

During anesthetic procedure, physiological variables were measured and recorded every 5 min and at six specific timepoints: T1, pre-incision; T2, after abdominal approach; T3, after right ovary excision; T4, after left ovary excision; T5, after uterus removal; and T6, after skin closure. Intraoperative monitoring includes HR, *f*_R_, inspired isoflurane concentration (FIIso), end-tidal carbon dioxide (PE’CO_2_), and oxygen saturation with pulse oximetry (SpO_2_) using a multiparametric monitor (BM7 VET Pro; Bionet Co., Ltd., Gunpo, Republic of Korea). Arterial blood pressure was measured using an appropriately sized cuff (width approximately 30–40% of the limb circumference) placed above the carpus.

### 2.4. Assessment of Postoperative Pain and Sedation

The scores of sedations and postoperative pain were assessed at 30, 60, 90, and 120 min after endotracheal extubation by a blinded, experienced pain assessor (W.C.). Pain scores were evaluated using CMPS-SF. Physiological variables were also evaluated, including HR, *f*_R_, and NIBP. If any dog reached a total CMPS-SF score of ≥5/20 at any postoperative timepoint, rescue analgesia was provided with intravenous fentanyl at a dose of 3 µg/kg. Pain scores were reassessed 5–10 min after administration. If pain scores subsequently decreased, an additional 0.5 mg/kg of subcutaneous morphine (Morphine sulfate injection, M&H Manufacturing, Samut Prakan, Thailand) was administered to ensure and maintain effective pain control. Pain scores obtained at the time of rescue were recorded; however, any scores collected afterward were excluded from statistical analysis and data evaluation. The dogs remained under observation until they fully recovered and were discharged.

At 120 min after extubation, all dogs received subcutaneous carprofen (Rimadyl^®^, Inovat Industry Pharmaceuticals Ltd., Sao Paulo, Brazil) at a dose of 4.4 mg/kg. Additionally, dogs were prescribed carprofen (Rimadyl^®^, Zoetis Inc., Lincoln, NE, USA) orally at a dose of 4.4 mg/kg once daily for seven consecutive days. On the day of suture removal, all dogs were checked and recorded for surgical wound complications.

### 2.5. Statistical Analyses

The sample size was calculated using a statistical formula for comparing two independent means. This calculation utilized postoperative CMPS-SF pain score data obtained two hours after OVH in dogs reported by Çolak and Yılmaz [36], based on the mean and standard deviation (pain scores of the control group: 4.08 ± 0.33 and the treatment group: 3.25 ± 0.18), with an effect size calculated at 0.83. The sample size calculation was performed with a statistical power of 0.80 and a significance level of 0.05, indicating that a sample size of 10 dogs per group was required.

Data were assessed for normal distribution and homogeneity of variance using Shapiro–Wilk and Levene’s tests, respectively. Preoperative variables (age, body weight, HR, *f*_R_, and NIBP), anesthetic duration, surgical duration, time to extubation, and wound length were compared between groups using one-way analysis of variance (ANOVA) for parametric data, and the Kruskal–Wallis test for nonparametric data. Intraoperative (HR, *f*_R_, NIBP, and FIIso) and postoperative variables (HR, *f*_R_, and NIBP) were descriptively reported. Postoperative pain scores of CMPS-SF and sedation scores were analyzed using a linear mixed model with repeated measurements. The dog was used as a random effect, while treatment groups, timepoints, and their interaction were considered fixed effects, using an unstructured variance component. Differences in outcomes between groups at each timepoint were analyzed using the CONTRAST options with Bonferroni adjustment. The number of rescue dogs across groups was analyzed for the differences in their proportions using Chi-square or Fisher’s exact test. Data are presented as mean ± standard deviation. All statistical analyses were conducted using STATA software (STATA statistical software version 14.1; StataCorp LLC, College Station, TX, USA). A *p* < 0.05 was considered statistically significant.

## 3. Results

Thirty healthy female dogs completed the study; 27 dogs were classified as ASA I and 3 dogs as ASA II. No significant differences were observed between groups for preoperative variables (age, body weight, HR, *f*_R_, SAP, MAP, and DAP), duration of anesthesia, duration of surgery, time to extubation, and wound length (Table 1).

Intraoperative (HR, *f*_R_, SAP, MAP, DAP, and FIIso) and postoperative variables (HR, *f*_R_, SAP, MAP, and DAP) were descriptively reported (Table 2 and Table 3). All vital signs were within normal ranges. The SpO_2_ values were above 95%, PE’CO_2_ ranged from 35 to 45 mmHg, and body temperature was between 37 and 38 °C in all dogs during surgery.

Postoperative pain scores assessed using the CMPS-SF in the C group were significantly higher than in the IV group at T60 (3.0 ± 2.8 vs. 1.6 ± 0.7, *p* = 0.004) and T120 (2.3 ± 0.8 vs. 1.4 ± 0.7, *p* = 0.004) and higher than in the IP + SP group at T60 (3.0 ± 2.8 vs. 1.2 ± 0.4, *p* = 0.004), T90 (1.7 ± 0.8 vs. 1.1 ± 0.3, *p* = 0.029), and T120 (2.3 ± 0.8 vs. 1.3 ± 0.5, *p* = 0.004), but no significant differences were found between the IP + SP and IV groups at any postoperative timepoints (Table 4). Sedation scores did not differ significantly between groups. No rescue analgesia was required in the IP + SP and IV groups. In the C group, three dogs were administered rescue analgesia at T30 (*n* = 1) and T60 (*n* = 2), which was significantly different compared to the IV and IP + SP groups (*p* = 0.03). After administration of rescue analgesia, pain scores in all dogs remained low until discharge. None of the dogs exhibited adverse effects of lidocaine throughout the study, and no complications were noted at the time of suture removal 10–14 days after surgery.

## 4. Discussion

In our present study, both intravenous lidocaine infusion and a combination of intraperitoneal and incisional blocks demonstrated effective early postoperative analgesia for up to 120 min after surgery, compared to the controls. Administration of intravenous lidocaine at a dose of 2 mg/kg, followed by a CRI of 50 μg/kg/min, resulted in effective early postoperative pain control. In veterinary medicine, lidocaine infusions during anesthesia have demonstrated analgesic benefits in dogs, administered as a loading dose of 1–2 mg/kg, followed by a CRI ranging from 25 to 200 μg/kg/min [12,31,37,38,39]. Pain scores, as assessed by the CMPS-SF, were significantly lower in the IV group compared to the C group at 60 and 120 min after extubation, and rescue analgesia was not required in any dogs in the IV group. The analgesic efficacy observed with intravenous lidocaine infusion in this study is consistent with previous studies in dogs undergoing surgery [12,37,39]. These findings suggested that intravenous lidocaine infusion may be an alternative for early postoperative analgesia in dogs undergoing OVH.

Locoregional anesthetic techniques for canine OVH exist in various approaches, including epidural, transversus abdominis plane, quadratus lumborum, and combinations of intraperitoneal with incisional (infiltration or splash methods) blocks [40,41,42]. Although a combination of intraperitoneal and incisional blocks is simple and recommended in the current pain management guidelines, their use in routine practice remains limited [43]. In contrast, intravenous lidocaine infusion is a well-established and technically straightforward approach, making it an attractive option for preemptive systemic analgesia in dogs. Moreover, postoperative CMPS-SF pain scores in the IV group were low, no rescue analgesia was required, and the scores did not differ significantly compared to the IP + SP groups up to 120 min. These findings indicate that the early postoperative analgesic efficacy of intravenous lidocaine infusion is comparable to the combination of intraperitoneal and incisional lidocaine splash block in dogs undergoing OVH.

Analgesic plasma concentrations of lidocaine in isoflurane-anesthetized dogs have been reported to range from 0.4 to 2.13 μg/mL when an intravenous loading dose of 1–2 mg/kg was followed by a CRI of 25–50 μg/kg/min [37,39]. Plasma concentrations associated with systemic toxicity have been reported to initiate seizures at an average of 11.2 μg/mL [44]; however, it is recognized that seizures represent a progression of central nervous system toxicity which may further escalate to cardiovascular collapse or death at even higher plasma concentrations. Although plasma lidocaine concentrations were not directly measured in our study, previous research using the same dosing protocol—a loading dose of 2 mg/kg followed by a CRI of 50 μg/kg/min—demonstrated plasma concentrations of approximately 1.54 μg/mL [31], a level well below established seizure-inducing thresholds. Consistent with previous reports, no seizures or other adverse effects were observed in the present study. These findings collectively support the efficacy and safe use of systemic lidocaine for analgesia in dogs undergoing surgery. However, errors in dose calculation and dilution may elevate the risk of adverse effects, emphasizing the need for an effective tool to ensure accurate and efficient drug preparation [45].

Systemic lidocaine has proven to elicit an anesthetic-sparing effect [20,25,26,27,28,29,30,31]. Intravenous lidocaine infusions are acceptable for use in anesthetized dogs as an adjunct to enhance the efficacy of balanced anesthesia in clinical practice [12]. Previous studies have reported that administering intravenous lidocaine at a dose of 2 mg/kg, followed by a CRI at 50 μg/kg/min, decreases the minimum alveolar concentration of isoflurane by 18.7% and helps to prevent the sympathetic response to surgical stimulation in clinically healthy dogs [12,31]. This is the same regimen used in the present study. However, the antinociceptive effect of systemic lidocaine remains debated [20,21,22]. Some studies have suggested that systemic lidocaine may provide anesthetic-sparing effects rather than true antinociception, as evidenced by observed mild to moderate sedative effects and prolonged recoveries after surgery [22,31]. In our present study, there were no significant differences in extubation times or sedation scores between groups. Although a numerical reduction in FIIso was observed in the IV group during surgery, which may be associated with an anesthetic-sparing effect. However, end-tidal isoflurane concentration is preferable and should be employed instead of FIIso. Therefore, further studies are warranted to confirm the antinociceptive effect of systemic lidocaine.

Opioids are potent analgesics recommended for perioperative pain management. In the present study, fentanyl was administered intravenously to all dogs prior to surgical incision as a preemptive analgesic strategy. In the C group, fentanyl was used as a sole analgesic modality, with no additional pain management techniques, whereas lidocaine was used as an adjunctive strategy in both treatment groups. Preemptive analgesic protocols have been shown to reduce postoperative pain [46]. Despite preemptive fentanyl administration, postoperative CMPS-SF pain scores were notably higher in the C group, with 3 out of 10 dogs requiring rescue analgesia, which significantly differed compared to the treatment group. These findings suggest that unimodal analgesia is insufficient for OVH, a procedure associated with moderate pain, resulting in inadequate analgesic control (oligoanalgesia). In contrast, both IV and IP + SP groups exhibited significantly lower pain scores. Accordingly, the implementation of multimodal analgesia is essential for optimal pain management and is recommended for surgical procedures.

To minimize bias, all surgical procedures were performed by the same experienced surgeon, thereby reducing variability in surgical trauma and subsequent nociceptive stimulation. Furthermore, pain assessment was performed by the same experienced observer who was unaware of the treatment group. The use of the CMPS-SF as a validated pain scoring system further supports the accuracy and consistency of our pain assessment and management in this study. Nevertheless, this study has several limitations. The inclusion of a negative control group (dogs treated with saline) would have strengthened the comparative analysis. However, for ethical reasons and the potential need for intraoperative rescue analgesia, all dogs—including those in the control group—received fentanyl as preemptive analgesia. Pain scores recorded after administration of rescue analgesia were excluded from the statistical analysis. Nonetheless, preemptive fentanyl and exclusion of data from dogs receiving rescue analgesia may have resulted in lower postoperative pain scores in the control group, thereby reducing the ability to detect differences between groups. Moreover, pain assessments were conducted for 120 min following surgery. After carprofen was administered subcutaneously at 120 min, dogs continued to be monitored until 180 min postoperatively, at which point monitoring was discontinued and the dogs were subsequently discharged to their owners. Optimal postoperative pain assessment should extend for at least four to six hours in dogs undergoing OVH [47,48]. Pain and potential complications in this study were not evaluated beyond this timepoint, longer-term follow-up is therefore recommended. In addition, the duration of analgesic efficacy should be assessed over a 24 h postoperative period using a multimodal assessment approach. Additionally, the use of end-tidal isoflurane concentration was not implemented in this study. Including real-time measurements of end-tidal isoflurane would allow for more precise anesthetic monitoring. In this study, both lidocaine IV and the combination of IP and SP blocks provided effective analgesia. It would be of interest to investigate these techniques in combination as part of a multimodal analgesic protocol. Further studies exploring the combination of these techniques, as well as various dosages and surgical procedures utilizing lidocaine infusion, are warranted to confirm and optimize perioperative pain management in dogs undergoing surgery.

## 5. Conclusions

Intravenous lidocaine infusion and the combination of lidocaine intraperitoneal and incisional splash blocks provided effective early postoperative analgesia for up to 120 min after extubation in dogs undergoing OVH. No significant differences in analgesic efficacy were found between these two techniques, and no adverse effects were observed. Therefore, intravenous lidocaine infusion may be considered as an option for early postoperative pain management when locoregional techniques are impractical or unavailable in dogs undergoing OVH.

## Figures and Tables

**Table 1:** Preoperative variables, duration of anesthesia and surgery, time to extubation, and wound length in lidocaine intraperitoneal splash combined with incisional splash block (IP + SP), intravenous lidocaine infusion (IV), and control (C) groups in dogs undergoing ovariohysterectomy.

Parameter	Group		
	IP + SP (*n* = 10)	IV (*n* = 10)	C (*n* = 10)
Age (months)	18.3 ± 15.0	22.3 ± 12.1	19.6 ± 12.6
Body weight (kg)	13.4 ± 6.0	13.7 ± 6.1	13.1 ± 5.0
HR (bpm)	121 ± 21	130 ± 30	116 ± 17
*f*_R_ (bpm)	58 ± 8	58 ± 8	58 ± 4
SAP (mmHg)	140 ± 16	137 ± 23	134 ± 17
MAP (mmHg)	102 ± 13	108 ± 19	103 ± 15
DAP (mmHg)	83 ± 14	94 ± 19	88 ± 16
Duration of anesthesia (min)	61.7 ± 8.2	55.6 ± 3.3	55.7 ± 6.5
Duration of surgery (min)	38.0 ± 5.4	34.1 ± 3.2	35.9 ± 3.8
Time to extubation (min)	10.4 ± 2.6	11.6 ± 5.4	9.0 ± 5.2
Wound length (cm)	5.1 ± 1.4	4.9 ± 1.2	5.7 ± 0.8

Data are represented as mean ± standard deviation. C, control; DAP, diastolic arterial pressure; *f*_R_, respiratory rate; HR, heart rate; IP + SP, lidocaine intraperitoneal splash combined with incisional splash block; IV, intravenous lidocaine infusion; MAP, mean arterial pressure; SAP, systolic arterial pressure.

**Table 2:** Intraoperative variables in the lidocaine intraperitoneal splash combined with incisional splash block (IP + SP; *n* = 10), intravenous lidocaine infusion (IV; *n* = 10), and control (C; *n* = 10) groups in dogs undergoing ovariohysterectomy.

Variable	Group	Timepoint					
		T1	T2	T3	T4	T5	T6
HR (bpm)	IP + SP	115 ± 17	97 ± 16	102 ± 21	107 ± 17	110 ± 18	113 ± 23
	IV	111 ± 18	96 ± 18	105 ± 19	100 ± 14	101 ± 13	106 ± 19
	C	124 ± 23	107 ± 13	104 ± 15	104 ± 10	104 ± 15	113 ± 18
*f*_R_ (bpm)	IP + SP	14 ± 2	11 ± 2	13 ± 2	11 ± 2	12 ± 3	15 ± 6
	IV	13 ± 5	11 ± 3	13 ± 5	13 ± 4	12 ± 5	13 ± 6
	C	14 ± 4	14 ± 5	18 ± 7	16 ± 7	13 ± 4	16 ± 5
SAP (mmHg)	IP + SP	104 ± 15	106 ± 14	119 ± 16	117 ± 22	105 ± 22	106 ± 17
	IV	107 ± 15	107 ± 14	122 ± 15	133 ± 25	115 ± 25	114 ± 17
	C	106 ± 18	115 ± 21	128 ± 22	129 ± 19	120 ± 15	123 ± 16
MAP (mmHg)	IP + SP	72 ± 14	78 ± 9	89 ± 15	88 ± 18	80 ± 23	77 ± 15
	IV	76 ± 20	81 ± 18	95 ± 15	102 ± 13	87 ± 18	88 ± 17
	C	75 ± 19	90 ± 21	103 ± 20	99 ± 13	95 ± 16	95 ± 17
	IP + SP	56 ± 14	64 ± 9	75 ± 15	74 ± 17	68 ± 24	62 ± 15
DAP (mmHg)	IV	61 ± 22	68 ± 21	81 ± 15	85 ± 13	73 ± 16	75 ± 18
	C	61 ± 20	77 ± 22	91 ± 20	84 ± 15	83 ± 17	79 ± 20
FIIso	IP + SP	2.1 ± 0.3	2.2 ± 0.8	2.5 ± 0.6	2.5 ± 0.6	2.3 ± 0.5	1.8 ± 0.4
	IV	2.1 ± 0.4	1.9 ± 0.5	2.5 ± 0.8	2.2 ± 0.5	1.9 ± 0.2	1.6 ± 0.3
	C	2.0 ± 0.6	2.2 ± 1.0	2.8 ± 0.9	2.6 ± 1.0	2.5 ± 1.0	1.8 ± 0.5

Data are represented as mean ± standard deviation. C, control; DAP, diastolic arterial pressure; FIIso, inspired isoflurane concentration; *f*_R_, respiratory rate; HR, heart rate; IP + SP, lidocaine intraperitoneal splash combined with incisional splash block; IV, intravenous lidocaine infusion; MAP, mean arterial pressure; SAP, systolic arterial pressure. T1, pre-incision; T2, after abdominal approach; T3, after right ovary excision; T4, after left ovary excision; T5, after uterus removal; and T6, after skin closure.

**Table 3:** Postoperative physiological variables in the lidocaine intraperitoneal splash combined with incisional splash block (IP + SP), intravenous lidocaine infusion (IV), and control (C) groups in dogs undergoing ovariohysterectomy.

Variable	Group	Timepoint			
		T30	T60	T90	T120
HR (bpm)	IP + SP	140 ± 32	121 ± 22	116 ± 29	112 ± 31
	IV	150 ± 36	137 ± 30	121 ± 29	116 ± 30
	C	131 ± 31	130 ± 24	119 ± 25	115 ± 24
*f*_R_ (bpm)	IP + SP	22 ± 4	23 ± 7	20 ± 5	22 ± 13
	IV	20 ± 6	19 ± 4	20 ± 7	19 ± 5
	C	30 ± 17	26 ± 14	25 ± 9	30 ± 12
SAP (mmHg)	IP + SP	131 ± 15	137 ± 17	128 ± 20	145 ± 19
	IV	135 ± 25	132 ± 17	140 ± 23	142 ± 16
	C	140 ± 18	148 ± 16	156 ± 15	143 ± 26
MAP (mmHg)	IP + SP	108 ± 16	108 ± 13	100 ± 15	113 ± 18
	IV	103 ± 12	106 ± 12	112 ± 16	112 ± 12
	C	112 ± 19	119 ± 16	117 ± 11	113 ± 21
DAP (mmHg)	IP + SP	97 ± 17	95 ± 12	86 ± 14	98 ± 20
	IV	87 ± 9	92 ± 12	97 ± 14	95 ± 18
	C	97 ± 21	104 ± 17	98 ± 14	98 ± 20

In the IP + SP and IV groups, *n* = 10 at all timepoints. In the control group, *n* = 10 at T30, *n* = 9 at T60, and *n* = 7 at T90 and T120. Data are represented as mean ± standard deviation. C, control; DAP, diastolic arterial pressure; *f*_R_, respiratory rate; HR, heart rate; IP + SP, lidocaine intraperitoneal splash combined with incisional splash block; IV, intravenous lidocaine infusion; MAP, mean arterial pressure; SAP, systolic arterial pressure. T30, 30 min after extubation; T60, 60 min after extubation; T90, 90 min after extubation; T120, 120 min after extubation.

**Table 4:** CMPS-SF pain scores and sedation scores in the lidocaine intraperitoneal splash combined with incisional splash block (IP + SP), intravenous lidocaine infusion (IV), and control (C) groups in dogs undergoing ovariohysterectomy.

Parameter	Group	Timepoint			
		T30	T60	T90	T120
CMPS-SF	IP + SP IV C	1.4 ± 0.7 1.7 ± 0.8 2.6 ± 2.5	1.2 ± 0.4 ^B^ 1.6 ± 0.7 ^B^ 3.0 ± 2.8 ^A^	1.1 ± 0.3 ^B^ 1.8 ± 1.0 ^AB^ 1.7 ± 0.8 ^A^	1.3 ± 0.5 ^B^ 1.4 ± 0.7 ^B^ 2.3 ± 0.8 ^A^
Sedation	IP + SP IV C	5.9 ± 3.2 5.9 ± 2.7 4.7 ± 1.8	4.4 ± 3.0 4.4 ± 2.3 3.6 ± 1.2	3.4 ± 2.4 2.9 ± 1.6 3.3 ± 1.8	3.4 ± 3.7 2.0 ± 1.1 3.9 ± 2.1

In the IP + SP and IV groups, *n* = 10 at all timepoints. In the control group, *n* = 10 at T30, *n* = 9 at T60, and *n* = 7 at T90 and T120. Data are represented as mean and standard deviation. Groups are labeled with superscript capital letters (^A, B^); groups with different superscript capital letters are significantly different (*p* = 0.004 at T60 and T120 and *p* = 0.029 at T90). C, control; CMPS-SF, Glasgow Composite Measure Pain Scale-Short Form; IP + SP, lidocaine intraperitoneal splash combined with incisional splash block; IV, intravenous lidocaine infusion. T30, 30 min after extubation; T60, 60 min after extubation; T90, 90 min after extubation; T120, 120 min after extubation.

## Data Availability

The original contributions presented in this study are included in the article. Further inquiries can be directed to the corresponding author.
